# Kinesin superfamily member KIFC2 as an independent prognostic biomarker of colon adenocarcinoma associated with poor immune response

**DOI:** 10.1097/MD.0000000000035491

**Published:** 2023-10-27

**Authors:** Tao Chen, Yunqian Chu, Haiyuan Xu, Hanjue Dai, Yuxi Zhou, Haiwei Du, Wenyu Zhu

**Affiliations:** a Department of Gastrointestinal Surgery, The Affiliated Changzhou No.2 People’s Hospital with Nanjing Medical University, Changzhou, Jiangsu, China; b Cancer Center, The Affiliated Changzhou No. 2 People’s Hospital with Nanjing Medical University, Changzhou, Jiangsu, China; c Department of Medical Oncology, Kunshan First People’s Hospital Affiliated to Jiangsu University, Kunshan, Jiangsu, China; d Burning Rock Biotech, Guangzhou, China.

**Keywords:** colon adenocarcinoma, colorectal cancer, KIFC2, kinesin, prognostic biomarker

## Abstract

Clinical outcomes of colon adenocarcinoma (COAD) exhibit heterogeneity among different patients, highlighting the need for novel prognostic biomarkers. Kinesin superfamily members have been shown to play a crucial role in tumors and can predict cancer diagnosis and prognosis. However, the role of kinesin family member C2 (KIFC2) in tumors, particularly its prognostic value in COAD, remains poorly understood. Our bioinformatics analysis of the cancer genome atlas and GEO databases revealed significantly higher expression of KIFC2 in COAD, correlating with a worse prognosis in the cancer genome atlas-COAD and GSE17536 cohorts. Additionally, differentially expressed genes in COAD were enriched in immune-related pathways, and patients with higher KIFC2 expression showed fewer activated CD4 + T cells. These findings suggest KIFC2 as a potential prognostic biomarker for COAD, warranting further validation in clinical studies.

## 1. Introduction

Colorectal cancer (CRC) is the third most common cancer and the second leading cause of cancer-related death worldwide.^[1]^ Colon adenocarcinoma (COAD) is the most common type of CRC given the fact that more than 90% of CRC were adenocarcinomas originating from epithelial cells of the colorectal mucosa.^[2]^ The prognosis of CRC was mainly dependent on the tumor stage at the time of diagnosis.^[3]^ The overall 5-year survival rates of CRC were approximately 90% for stage I, 70% for stage II, 58% for stage III, and <15% for stage IV diseases, respectively.^[3]^ During clinical practice, patients with the same tumor stage may have varied prognoses as CRC is highly complex and heterogenous.^[4]^ Therefore, it remains an unmet clinical need to discover and validate novel prognostic biomarkers which could estimate disease progression or OS of CRC patients.

Advances in high-throughput genomic techniques such as microarray and next-generation sequencing contributed to a more comprehensive understanding of CRC at the molecular level.^[5]^ In addition to conventional clinicopathological factors, molecular features such as gene mutation, expression and DNA methylation also play important roles in underlying tumorigenesis and tumor progression.^[4,5]^ More recently, genomic databases such as TCGA (the cancer genome atlas) and GEO (gene expression omnibus) which included relatively large-scale genomic data of different cancer types have been used as a valuable source to explore novel biomarkers of CRC.^[6–8]^

Kinesin family member C2 (KIFC2) is a member of kinesin superfamily proteins,^[9]^ which function as transporters along microtubules during intracellular trafficking of functional proteins, organelles and biomacromolecules.^[10]^ Several KIF genes have been identified to play crucial roles in tumors and predict cancer diagnosis and prognosis.^[11–13]^ KIFC2 is highly expressed in various cancers, including colon cancer. Prior research has suggested its importance in dendrite development.^[14]^ However, the clinical predictive value of KIFC2 remains relatively unexplored. Chen et al^[15]^ found KIFC2 overexpression in hepatocellular carcinoma associated with shorter overall survival (OS) times, though not confirmed in multivariate analysis. Another recent study^[16]^ showed significantly higher KIFC2 gene expression in CRPC samples compared to normal and PCa samples, with higher expression linked to poorer progression-free interval. Nonetheless, the association of KIFC2 expression levels with the prognosis of patients with colon cancer remains unclear.

In the present study, we used datasets obtained from TCGA and GEO to evaluate the expression of KIFC2 in COAD. The role of KIFC2 expression as an independent prognostic factor of COAD was discovered in the TCGA-COAD dataset and validated in a GEO dataset. Further bioinformatic analysis^[17,18]^ of genes and pathways related to KIFC2 expression was performed to understand the role of KIFC2 in COAD. Identifying patients with different prognoses through informativae biomarkers might contribute to better clinical management of COAD.

## 2. Methods

### 2.1. Data collection

The TCGA data were obtained from the UCSC Xena website (http://xena.ucsc.edu/).^[6,19]^ The tumor immune estimation resource was used to analyze KIFC2 expression levels between tumor and normal tissues in TCGA dataset.^[20]^ The GSE17536 dataset was obtained from the GEO website (https://www.ncbi.nlm.nih.gov/geo).

### 2.2. Construction of related genes network

The gene-gene functional interaction network of KIFC2 was constructed by GeneMANIA 3.6.0 (http://www.genemania.org).^[21]^ The advanced statistical options were set as below: max resultant attributes = 10, max resultant genes = 20, and the weighing method used = automatically selected.

### 2.3. Gene set enrichment analysis (GSEA)

First, we ranked all the mRNAs according to the Fold Change between KIFC2 high and low expression groups. Then the ordered mRNAs were imported to the R package “clusterProfiler” (version 3.18.1) for GSEA analysis, containing KEGG (Kyoto encyclopedia of genes and genomes) pathways from Molecular Signatures Database (MSigDB) (https://software.broadinstitute.org/gsea/msigdb).

### 2.4. Identification of hub genes

The interaction between proteins was evaluated by the STRING (The Search Tool for the Retrieval of Interacting Genes) database (http://string.embl.de/).^[22]^ The minimum required interaction score was set as 0.4 to construct a PPI network. Cytoscape software (version 3.8.0) was subsequently used to visualize the PPI network.^[23]^ The cytoHubba plug-in was employed to discover the hub genes using the maximal clique centrality method. In addition, the correlation module of tumor immune estimation resource 2 was used to show the correlation between KIFC2 and hub genes. Enrichment analysis of these hub genes was performed using the R package “clusterProfiler” (version 3.18.1).

### 2.5. Statistical analysis

Kruskal–Wallis test was used to compare gene expression in different samples. Associations between gene expression and other clinical or molecular characteristics were evaluated by the Wilcoxon rank sum test for continuous variables or the Chi-square test for categorical variables. Kaplan–Meier method was applied for survival analysis and the log-rank test was used to estimate statistical significance. Multivariate Cox regression analysis was used to screen potential prognostic factors. The association of KIFC2 and immune microenvironment was investigated in the TCGA-COAD dataset. The above analysis was performed in R software (version 4.0.2, https://www.r-project.org/). All statistical tests are 2-tailed, and a *P* value < .05 was considered statistically significant.

## 3. Results

### 3.1. High expression levels of KIFC2 in COAD

**Figure 1.**
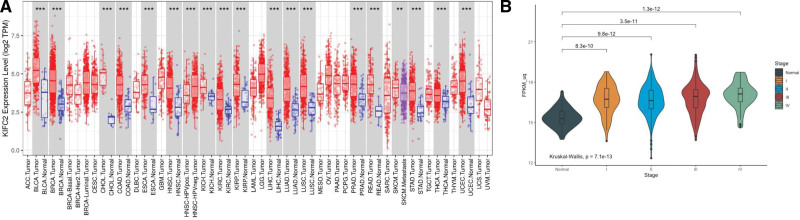
Expression of kinesin family member C2 (KIFC2) in all cancer types (A) and in different COAD stages (B). COAD (colon adenocarcinoma), READ (rectum adenocarcinoma), BLCA (bladder urothelial carcinoma), BRCA (breast invasive carcinoma), CHOL (cholangiocarcinoma), ESCA (esophageal carcinoma), HNSC (head and neck squamous cell carcinoma), KICH (kidney chromophobe), KIRC (kidney renal clear cell carcinoma), KIRP (kidney renal papillary cell carcinoma), LIHC (liver hepatocellular carcinoma), LUAD (lung adenocarcinoma), LUSC (lung squamous cell carcinoma), PRAD (prostate adenocarcinoma), STAD (stomach adenocarcinoma), THCA (thyroid carcinoma), UCEC (uterine corpus endometrial carcinoma), TPM (transcripts per million), FPKM (fragments per kilobase of transcript per million fragments mapped). *P < .05, **P < .01, ***P < .001.

In the TCGA dataset, expression levels of KIFC2 varied among different cancer types. Compared with corresponding normal tissue, KIFC2 showed significantly higher (*P* < .05) expression levels in COAD, rectum adenocarcinoma and other cancer types (Fig. 1A).

In the TCGA-COAD dataset, KIFC2 expression levels in 450 COAD patients were compared with 93 normal controls. There were 75 patients in stage I, 175 patients in stage II, 126 patients in stage III and 63 patients in stage IV. The KIFC2 expression levels were comparable (*P* > .05) in different stages of COAD. Significantly higher (*P* < .05) KIFC2 expression levels were consistently observed in each COAD stage than in normal tissues (Fig. 1B). The upper 25% expression level of KIFC2 was considered as high expression (n = 111) while the remaining lower 75% was considered as low expression (n = 338). The tumor mutation burden levels and microsatellite instability (MSI) status between COAD patients with high and low KIFC2 expression levels were also comparable (*P* > .05, data not shown).

### 3.2. Prognostic analysis of KIFC2 expression in COAD

**Figure 2.**
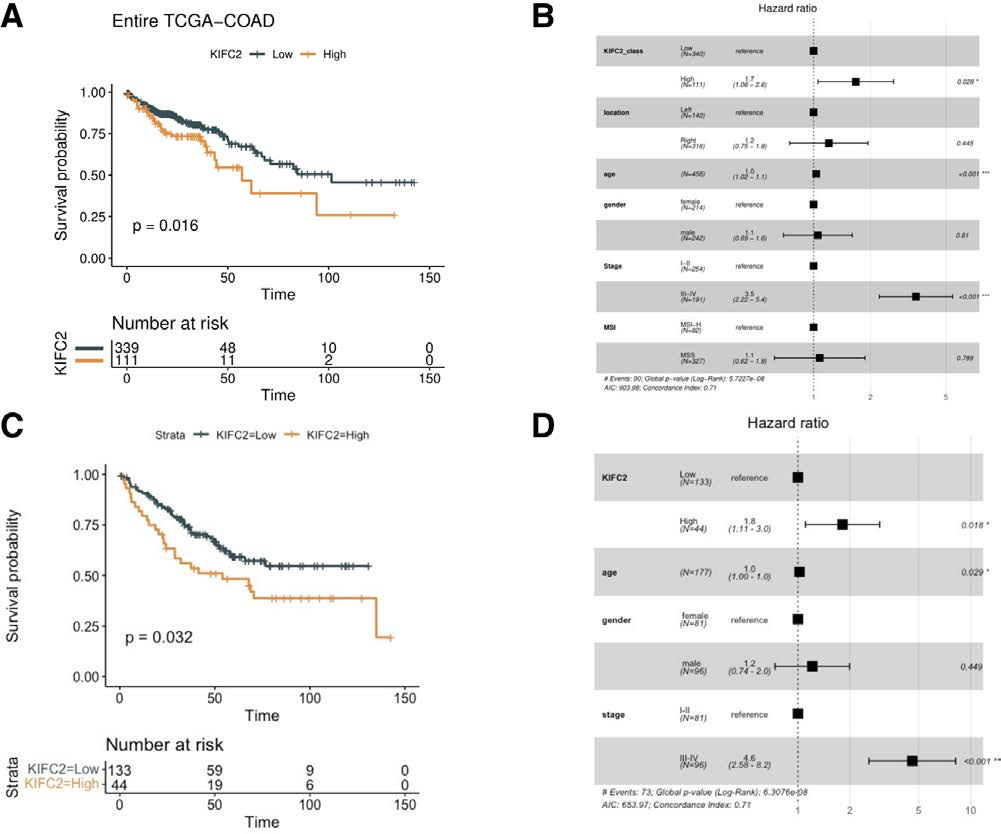
Kinesin family member C2 (KIFC2) expression and colon adenocarcinoma (COAD) prognosis in TCGA-COAD dataset (A and B), and in GSE17536 dataset (C and D). TCGA = the cancer genome atlas.

In the TCGA-COAD dataset, patients with high KIFC2 expression showed a significantly worse prognosis than the low expression group (hazard ratio [HR] = 1.672, *P* = .016, Fig. 2A). In COAD patients with MSI-H, an association between high KIFC2 expression and worse prognosis was also observed (HR = 3.139, *P* = .031) (Fig. S1A [Supplemental Digital Content, http://links.lww.com/MD/K200, which illustrates KIFC2 expression and COAD prognosis in patients with MSI and MSS]). In another subgroup of MSS COAD, patients with high KIFC2 expression showed a trend to worse prognosis but the significance threshold was not achieved (*P* = .099) (Fig. S1B, Supplemental Digital Content, http://links.lww.com/MD/K200, which illustrates KIFC2 expression and COAD prognosis in patients with MSI and MSS). When considering other factors related to COAD such as age, gender, tumor location, stage and MSI status, high expression of KIFC2 remained an independent risk factor for worse prognosis during multivariate analysis (HR = 1.7, *P* = .028, Fig. 2B).

The prognostic role of KIFC2 expression in COAD was also validated in the dataset GSE17536 obtained from GEO. According to the KIFC2 expression cutoff used in the TCGA-COAD dataset (upper 25% vs lower 75%), there were 44 patients with high expression and 133 patients with low expression in GSE17536. High expression of KIFC2 was significantly associated with a worse prognosis in COAD (HR = 1.69, *P* = .032, Fig. 2C). During multivariate analysis involving age, gender, and tumor stage, high expression of KIFC2 was also significantly associated with a worse prognosis of COAD (HR = 1.8, *P* = .018, Fig. 2D).

### 3.3. Analysis of genes and pathways related to KIFC2 expression

**Figure 3.**
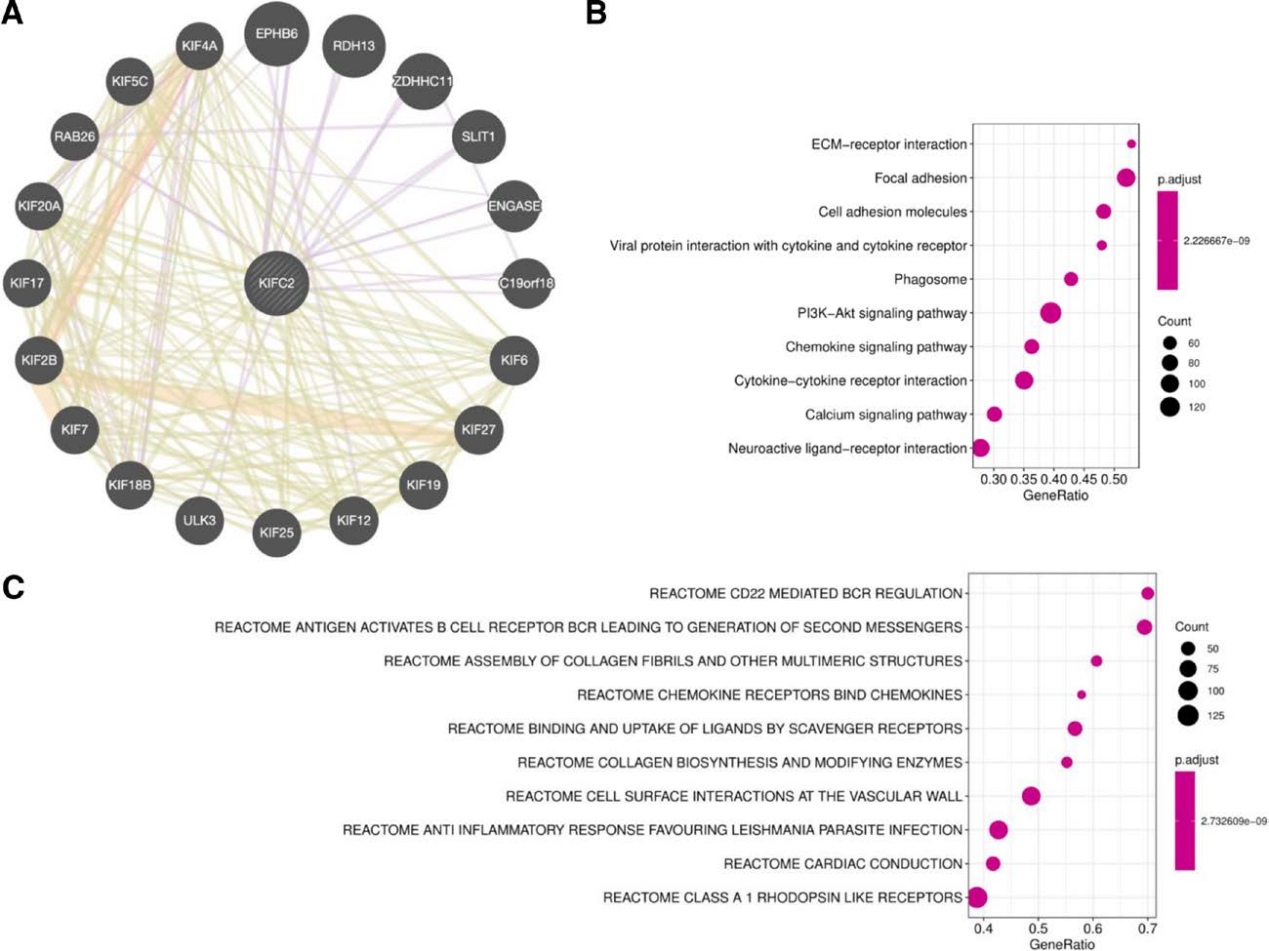
Analysis of genes and pathways related to kinesin family member C2 (KIFC2) expression by gene-gene functional interaction network (A), GSEA in KEGG pathways (B) and in REACTOME pathways (C). GSEA = gene set enrichment analsysis, KEGG = Kyoto encyclopedia of genes and genomes.

The gene-gene functional interaction network was constructed through GeneMANIA. A total of 20 genes related to KIFC2 were identified while 12 of them were KIF genes (Fig. 3A). However, linear correlations were not observed between the expression of KIFC2 and other KIF genes. To further understand the role of KIFC2 in COAD, differentially expressed genes (DEGs) were identified between high and low KIFC2 expression groups. The GSEA indicated that a considerable amount of DEGs was enriched in immune-related pathways such as viral protein interaction with cytokine and cytokine receptor, chemokine signaling pathway and cytokine-cytokine receptor interaction in KEGG (Fig. 3B) and CD22 mediated BCR regulation, antigen activates BCR leading to generation of second messengers, and chemokine receptors bind chemokines in REACTOME (Fig. 3C).

**Figure 4.**
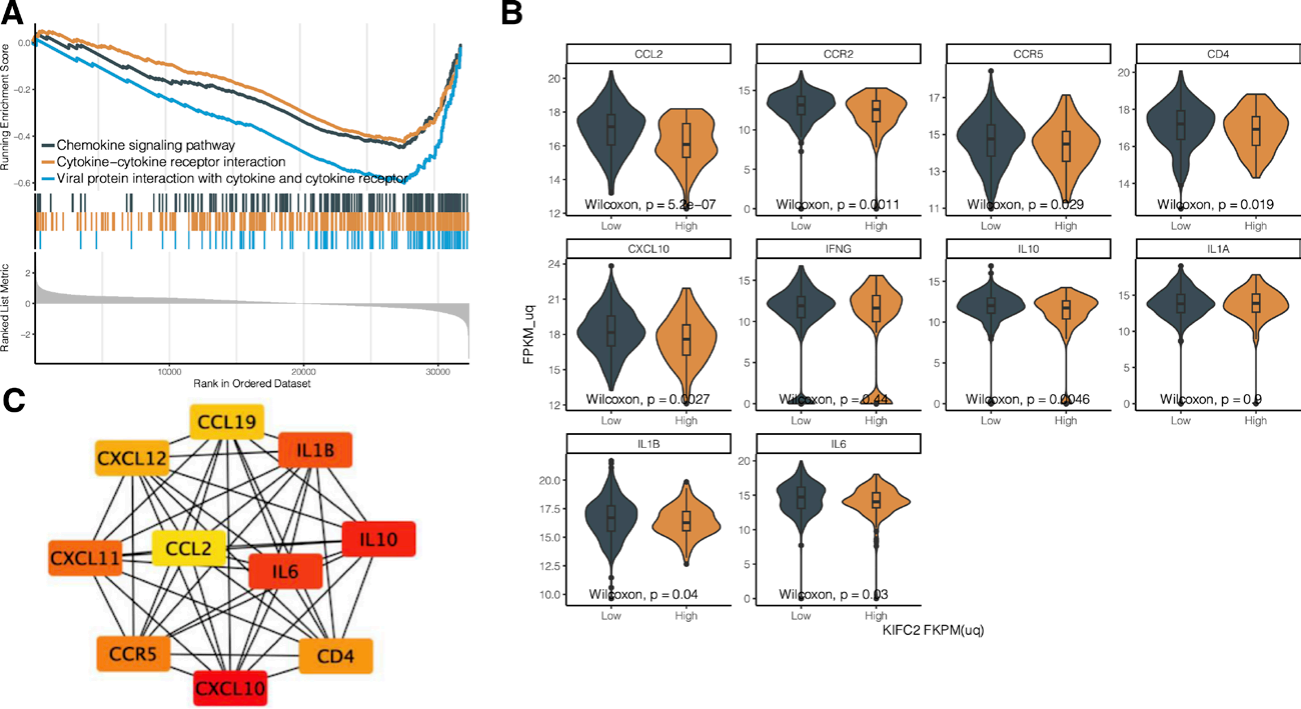
Analysis of hub genes in cytokine-cytokine receptor interaction pathway. (A) Down-regulation of 3 immune-related pathways in Kyoto encyclopedia of genes and genomes (KEGG). (B) Identification of top ten hub genes related to kinesin family member C2 (KIFC2) expression. (C) Expression of hub genes in different KIFC2 expression groups. FPKM (fragments per kilobase of transcript per million fragments mapped).

Our study also observed down-regulation of 3 immune-related pathways in KEGG (Fig. 4A). In the cytokine-cytokine receptor interaction pathway, there were a total of 102 genes which covered all genes in viral protein interaction with cytokine and cytokine receptor pathway and most genes in the chemokine signaling pathway. Therefore, further analysis was performed in the cytokine-cytokine receptor interaction pathway and identified the top ten hub genes including CCL19, CCL2, CCR5, CD4, CXCL10, CXCL11, CXCL12, IL10, IL1B, and IL6 (Fig. 4B). The expression levels in the 9 hub genes except CCL19 were negatively associated with the KIFC2 expression level (*P* < .05, Fig. 4B). The CCL19 gene showed the trend of higher expression in COAD patients with low KIFC2 expression but not achieved a significance threshold (*P* > .05, Fig. 4C).

### 3.4. Immune microenvironment analysis

**Figure 5.**
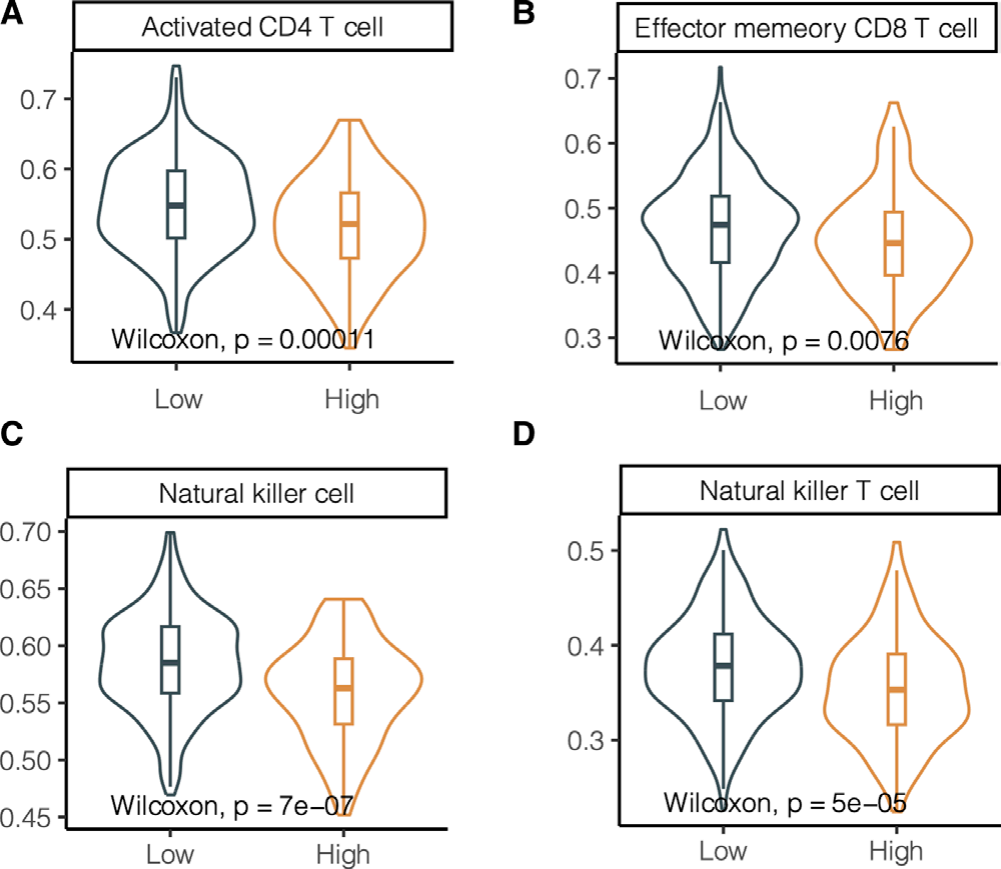
The association of KIF2C expression with immune environment in the TCGA-COAD dataset. (A) CD4 + T cells, (B) effector memory CD8 + T cells, (C) natural killer cells, (D) natural killer T cells. COAD = colon adenocarcinoma, TCGA = the cancer genome atlas.

Next, whether the KIFC2 expression impact on tumor microenvironment was explored. We found that patients with high KIFC2 expression had significantly lower levels of activated CD4^+^ T cells (Fig. 5A), effector memory CD8^+^ T cells (Fig. 5B), natural killer cells (Fig. 5C), and natural killer T cells (Fig. 5D). These data indicated that high expression associated with poor prognosis of COAD might attribute to less tumor-infiltrating cells and poor immune microenvironment.

## 4. Discussion

Gene expression has been widely used in the discovery of prognostic biomarkers of CRC.^[4]^ The expression levels of several genes such as PD-L1, STC2, LAYN, ApoE, and ZYX showed associations with poor OS of CRC.^[4]^ Our study focused on KIFC2, a member of the kinesin-14 family, and performed an integrative bioinformatic analysis in TCGA and GEO datasets. This gene showed higher expression levels in various cancer types including COAD. Significant associations between KIFC2 expression and survival probabilities were observed in the TCGA-COAD dataset and validated in a GEO dataset which suggested that a higher expression level of KIFC2 is indicative of poor prognosis in COAD. Numerous studies have explored the association between MSI and CRC prognosis. It has been consistently shown that patients with MSI-H tumors exhibit a better prognosis.^[24–26]^ In our study, we found that high expression of KIFC2 is an independent risk factor for worse prognosis in COAD, regardless of other factors such as MSI status, tumor location, and stage.

The KIFC2 gene, located in chromosome 8q24.3, encodes a neuron-specific C-terminal type KIF for dendritic transport of multivesicular body-like organelles.^[27,28]^ Previous studies reported that dysregulated kinesin expression and function were involved in tumorigenesis and metastasis of several cancer types, including breast, lung and colon cancer.^[29]^ In CRC, expression levels of KIF14, KIF18A, KIF20A, KIF4A, and KIF20B showed associations with tumor progression and prognosis by regulating cell survival, cell cycle, EMT(epithelial-mesenchymal transition) and microtubule dynamics.^[29]^ Meanwhile, KIF11, KIF22, KIF26B, and KIF2A also contribute to the development of CRC carcinogenesis.^[29]^Prior research indicates KIFC2 main expression in adult neurons and its association with MVB-like organelles.^[30]^ Its downregulation affects neuronal dendrite numbers, emphasizing KIFC2 role in dendrite development.^[27]^ Li team dual-gene model for CRPC includes KIFC2 and showed its elevated expression in CRPC samples and the progression group.^[16]^ However, there no established link between KIFC2 and CRC progression. The role of KIFC2 underlying CRC and other cancer types remains undefined. The tumor microenvironment, especially the immune aspect, profoundly impacts cancer outcomes and immune evasion.^[31]^ In our study, we constructed a gene-gene functional interaction network and identified a number of KIF genes that could interact with KIFC2 including KIF6, KIF27, KIF19, KIF12, KIF25, KIF18B, KIF7, KIF2B, KIF17, KIF20A, KIF5C, and KIF4A.

To further investigate the underlying mechanisms of KIFC2 in COAD, we identified numbers of DEGs between COAD patients with high and low KIFC2 expression. These genes were enriched in several immune-related pathways, in particular, the cytokine-cytokine receptor interaction pathway. KEGG analysis shows DEGs are mainly linked to cytokine-cytokine receptor interaction signaling pathway, hinting at their role in immune processes and COAD progression. We intensified our exploration of the relationship between hub genes and prognosis. We identified 2 hub genes in the chemokine signaling pathway, 3 in the viral protein interaction with cytokine and cytokine receptor signaling pathway, and 9 in thecytokine-cytokine receptor interaction signaling pathway that show significant prognostic differences based on their expression levels. CXCL8 is a common hub gene we identified among the 3 immune-related signaling pathways. In our study, high expression of CXCL8 is associated with a favorable prognosis of COAD (data not shown). This finding, however, contrasts with prior knowledge that suggests the expression of CXCL8 protein correlates with poor prognosis in CRC. For instance, literature reports correlate the mRNA levels of CXCL8 in tumoral tissues with CRC prognosis, OS, and tumor grade.^[32,33]^ CXCL8 is a hub gene intrinsically linked to CRC carcinogenesis, participating in various stages of its progression and metastasis. By binding its receptors, CXCL8 accelerates CRC cell proliferation, invasion, migration, and angiogenesis through the activation of the PIK3/Akt, MAPK, STAT3, and ERK1/2 signaling pathways.^[34]^ ACKR4, on the other hand, is a shared hub gene in the viral protein interaction with cytokine and cytokine receptor and cytokine-cytokine receptor interaction signaling pathways. We found that high expression of ACKR4 correlates with a good prognosis (data not shown). Studies have shown that a loss of ACKR4 in CRC is tied to reduced immune infiltration in the tumor microenvironment. More significantly, compared to stromal cells, the loss of ACKR4 in CRC tumor cells inhibits dendritic cell migration and antigen presentation to the tumor-draining lymph nodes. Additionally, tumors with ACKR4 knockdown become less receptive to immune checkpoint blockade.^[35]^ Associations of KIFC2 expression with expressions of hub genes including CCL19, CCL2, CCR5, CD4, CXCL10, CXCL11, CXCL12, IL10, IL1B, and IL6 were also observed. Recently, a group of chemotactic cytokines such as CCL2, CCL3, CCL5, CXCL1, CXCL2, and CXCL8 was reported to be involved in the initiation and progression of colon cancer.^[36]^ Another chemokine CCL20 was reported to have effects on fibroblasts, macrophages and immune cells which could influence the colon cancer tumor microenvironment.^[36]^ It was believed that CCL20 and its receptor CCR6 were involved in colon cancer progression through their interaction with several cytokines and toll-like receptors to increase aggressiveness.^[36]^ Therefore, the association between KIFC2 and the prognosis of CRC observed in our study might also be explained by immune activities and cytokines. The immune cells composition in the tumor environment significantly influences prognosis and therapy response.^[37]^ Different lymphocyte types can both help and hinder disease progression.^[38,39]^ Some recent colon cancer therapies failed due to low immune cell infiltration.^[40]^ Our data emphasize the association between identified kinesins and CD4 + and CD8 + T cells, key in antitumor responses. These cells detect tumor antigens on MHC Class I molecules and can directly kill cancer cells.^[41]^ Given the current limitation of lacking experimental validation, it is evident that further research is necessary to explore the interaction between KIFC2 and other genes. Addressing this aspect will be a key focus in our future investigations.

Considering the role of several kinesins underlying tumorigenesis and progression of CRC, it seems to be a promising approach to develop novel anticancer treatments by targeting kinesin superfamily members. Inhibition of kinesin spindle protein (KSP), encoded by the KIF11 gene, resulted in cell cycle arrest at mitosis with the formation of monogastric microtubule arrays, and ultimately in cell death.^[42]^ Although a number of KSP inhibitors have been investigated, their in vivo efficacies were unsatisfactory.^[43]^ Filanesib (Arry-520) is the only promising candidate which showed some promising results when used in combination with other anticancer drugs, especially against hematological malignancies.^[43]^ Despite strong preclinical rationales, the future development of KSP inhibitors as cancer treatments is still full of challenges.

Besides, we used the pRRophetic R package to predict drug susceptibility. Our findings suggest a strong correlation between KIFC2 expression and drug sensitivity (data not shown). Specifically, KIFC2 expression showed a positive correlation with the sensitivity to several drugs, including Axitinib, Crizotinib, Dabrafenib, Foretinib, Imatinib, Masitinib, Pazopanib, Ruxolitinib, Sunitinib, and Tivozanib. Conversely, it displayed a negative correlation with the sensitivity to Lisitinib (*P* < .001). It is worth mentioning that the expression of KIFC2 is also positively correlated with the sensitivity to 5-FU, although the correlation is not as strong (*P* = .038). Among these, the use of BRAF inhibitors, including Dabrafenib, for combined therapy has shown promising prospects.^[44,45]^ These results point towards KIFC2 as a valuable reference for selecting clinical anticancer drugs.

## 5. Conclusions

In conclusion, our study performed integrative bioinformatic analysis of KIFC2 expression in TCGA-COAD and GEO datasets and identified high expression of KIFC2 as an independent factor in predicting the poor prognosis of COAD. The molecular mechanisms underlying KIFC2 and COAD might associate with immune activities and cytokines. Further validation in clinical studies and functional investigations of KIFC2 expression are warranted.

## Author contributions

**Conceptualization:** Wenyu Zhu.

**Data curation:** Tao Chen, Yunqian Chu, Haiyuan Xu, Yuxi Zhou, Hanjue Dai, Haiwei Du, Wenyu Zhu.

**Formal analysis:** Tao Chen, Yunqian Chu, Haiyuan Xu, Yuxi Zhou, Haiwei Du, Wenyu Zhu.

**Investigation:** Tao Chen, Yunqian Chu, Haiyuan Xu.

**Methodology:** Yunqian Chu, Hanjue Dai.

**Project administration:** Wenyu Zhu.

**Software:** Tao Chen, Yunqian Chu, Haiyuan Xu, Hanjue Dai, Wenyu Zhu.

**Supervision:** Wenyu Zhu.

**Validation:** Tao Chen, Haiyuan Xu, Yuxi Zhou, Hanjue Dai.

**Visualization:** Tao Chen, Yuxi Zhou, Haiwei Du.

**Writing – original draft:** Tao Chen, Yunqian Chu, Haiyuan Xu, Yuxi Zhou, Haiwei Du, Wenyu Zhu.

**Writing – review & editing:** Haiyuan Xu, Yuxi Zhou, Haiwei Du, Wenyu Zhu.

## Supplementary Material


