# Supplementary figures and images for: Kinesin superfamily member KIFC2 as an independent prognostic biomarker of colon adenocarcinoma associated with poor immune response
Source: Medicine (Baltimore). 2023 Oct 27;102(43):e35491. doi: 10.1097/MD.0000000000035491 (PMC10615560; doi:10.1097/MD.0000000000035491)

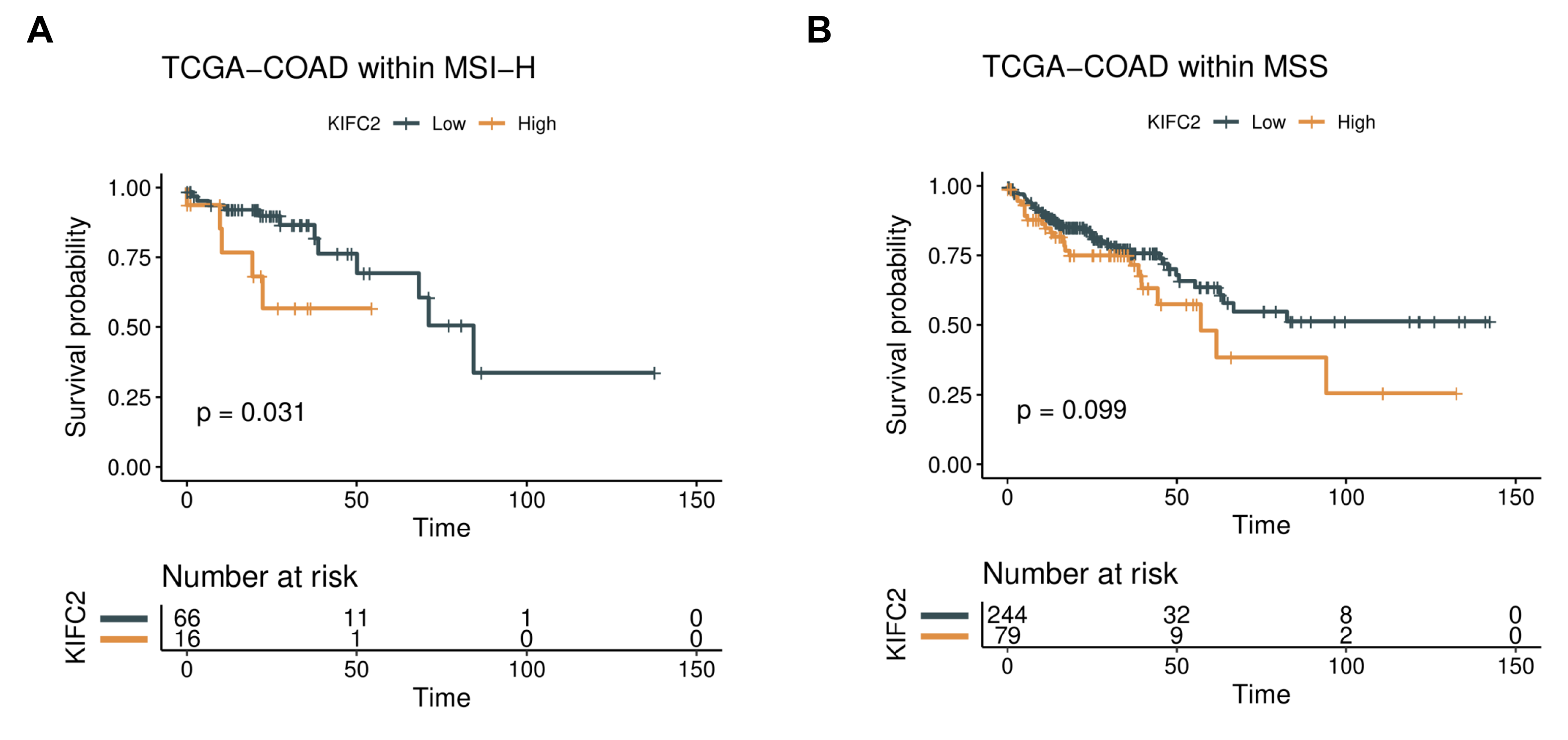

Supplement: Supplementary file 1 [file medi-102-e35491-s001.tif]
